# Corona virus and one step backward

**DOI:** 10.18502/ijm.v12i4.3942s

**Published:** 2020-08

**Authors:** Vahid Basirat, Seyed Farshad Allameh

**Affiliations:** Department of Gastroenterology, Imam Khomeini Hospital, Tehran University of Medical Sciences, Tehran, Iran

In ancient times, due to inaccessibility of human society to healthy water and food, the focus of health systems around the world especially in underdeveloped and developing countries were on infectious diseases and that is why we see a variety of vaccines and antibiotics today.

In a recent era of technological world and complexity of urban life, a lot of people used to new way of living including sedentary one and as a result of that recently increase in obesity and diseases like cardiovascular and diabetes should have been anticipated as we see nowadays. As far as these afore mentioned type of diseases have become commonplace, not surprisingly, the focus of countries’ health policies have been put on diagnosing and treating such disorders.

Moreover, infectious diseases have plagued humans throughout history and changing in behavior of human being, such as turning to unnatural food sources such as new animals, increased the risk of population being exposed to new viruses and microbes and we have forgotten that these contagious diseases have not only not been eliminated, but also increasingly endangering us, given changing in behavior and increase in communication between societies and so we are witnessing a resurgence of some extinct disease like polio in some countries and emergence of new ones like SARS and Covid-19 in others which by themselves have political, economic and social burden on all societies.

According to WHO statistics, about 13.3 million deaths occurred in 1998 as a result of infectious diseases which includes 25 percent of all causes of deaths. This number is even higher when it comes to low-income countries, accounting for about 45 percent of all causes of deaths and even higher among children with 63 percent of cases respectively (1) which partly can be prevented by allocating country’s resources on health policies and focusing on health issues.

As far as Imam Khomeini Hospital is concerned where we work in, there are a few things to keep in mind:

First of all with the start of Corona virus pandemic and due to fear that was created among people, a lot of patients had been rushed in to our hospital which is a tertiary care hospital and due to the nature of this virus, we had to consider an emergency department separately to isolate such patients with strong protocols and yet, although the disease is largely controlled, this fear has made our physicians to have protective equipment more than what World Health Organization (WHO) mentions.

Secondly, one of our major problems was that this virus is transmitted in droplets and it was important for us which procedures were aerosol producing and which types of Personal Protective Equipment (PPE) should be used in different procedures. We tried to identify them based on WHO protocols and distribute them among wards based on that.

The next problem that came up was that given the unknown nature of this virus and its way of transmission, should the pathway of these patients be separated from other patients or not and we made this separate way for them.

Another issue was the existence of various protocols to treat this virus. For example treatment protocols were Hydroxychloroquine, Oseltamivir, Lopinavir/Ritonavir or Atazanavir. Oseltamivir was removed due to its low effectiveness. Lopinavir/Ritonavir usage has been halted as it resulted in nausea and vomiting in majority of patients and instead Atazanavir was introduced in treatment regimen and even in end stage patients Corticosteroid, Intravenous immunoglobulin and Dialysis have been applied by some physicians as salvage therapy.

Next point was the pattern of involvement which was higher in men and less in children and made us to worked on and deal with and manage it in a proper way.

Moreover, one major issue of concern was that Corona virus targets multi internal organs in the body (2) so that it can involves different specialties and understanding this point is important for future strategies and decisions making.

Laboratory diagnostic tests as serologic test recently developed and the hope for producing vaccine for this virus become more reachable.

Last but not least is that this viral disease seems to be able to change all serious environmental conditions and life style of not only people of our country but also of the world have been changed after the onset of this disease and this by itself shows us the importance of going one step backward and fighting against infectious diseases along with paying attention to non-communicable diseases.

All in all and in the context of what has been noted above, the need for emergent reconsideration and change in health and economic priorities and policies of governments in all countries, improvement in laboratory diagnosis and application of new technology for rapid diagnosis of infectious diseases as well as strengthening research programs on vaccine development seems vital and necessary and we recommend that this consideration be taken to account in all important health meetings around the world.
